# COVID-19-Impfungen im Maßregelvollzug: einige Überlegungen zu ethischen und juristischen Aspekten

**DOI:** 10.1007/s00115-021-01212-y

**Published:** 2021-10-13

**Authors:** Peter Praus, Eva Biebinger, Harald Dreßing

**Affiliations:** 1 Klinik für Psychiatrie und Psychotherapie, J5, Zentralinstitut für Seelische Gesundheit, 68159 Mannheim, Deutschland; 2 Klinik für Forensische Psychiatrie, Pfalzklinikum für Neurologie und Psychiatrie, Weinstraße 100, 76889 Klingenmünster, Deutschland

## Hintergrund

Ohne Frage verbinden sich mit der zunehmenden Inanspruchnahme wirksamer Impfungen gegen COVID-19 Hoffnungen auf ein absehbares Ende der Coronaviruspandemie und den damit einhergehenden Einschränkungen von Grundrechten. Gerade in Kliniken des Maßregelvollzuges haben letztere im Interesse des Infektionsschutzes zu teils erheblichen Veränderungen der Behandlungsabläufe, der Durchführung von Lockerungen und somit – vor dem Hintergrund des Verhältnismäßigkeitsgrundsatzes – sowohl juristischen als auch ethischen Dilemmata geführt [15, 21]. Mittlerweile liegen nun, beruhend auf den Erfahrungen des bayerischen Maßregelvollzuges, belastbare, strukturierte Handlungsempfehlungen vor, die sowohl Infektionsschutz als auch Patientenrechte und Sicherheitsaspekte umfassend berücksichtigen und auf die für das Management künftiger Pandemien im Maßregelvollzug zurückgegriffen werden kann [8]. Daten aus dem Strafvollzug der Vereinigten Staaten von Amerika sowie aus Gefängnissen in England und Wales legen übereinstimmend nahe, dass die COVID-19-bedingte Mortalität in den jeweiligen Einrichtungen gegenüber der COVID-19-Mortalität in der Allgemeinbevölkerung in etwa um den Faktor 3 erhöht war [2, 19], was die enorme Bedeutung derartiger Maßnahmen in Einrichtungen des Straf- und Maßregelvollzuges unterstreicht. Aktuelle Daten aus dem Strafvollzug in Rhode Island, Vereinigte Staaten von Amerika, zeigen, dass mit den aktuell verfügbaren Impfstoffen bei gleichzeitig nur sehr selten beobachteten „Impfdurchbrüchen“ diesem dramatischen Befund abgeholfen werden könnte [3]. Nachvollziehbar ist somit auch, dass sowohl auf ethischer Grundlage als auch im Interesse des Infektionsschutzes eine Priorisierung inhaftierter bzw. untergebrachter Personen im Zuge nationaler Impfkampagnen gegen COVID-19 geboten ist [7, 20].

### Situation in der Bundesrepublik Deutschland

Während der Deutsche Ethikrat bereits im Februar 2021 Überlegungen zu der Frage veröffentlichte, wie der mögliche Wegfall der Gründe für den Entzug individueller Freiheitsrechte infolge einer wirksamen Impfung unter normativen Gesichtspunkten mit dem Bedürfnis der Allgemeinheit nach Schutz vor einer COVID-19-Infektion und Gleichbehandlung in Einklang gebracht werden könnte, wurden auch explizit marginalisierte, in besonderem Maße schutzbedürftige Gruppen in die o. g. Überlegungen mit einbezogen (https://www.ethikrat.org/fileadmin/Publikationen/Ad-hoc-Empfehlungen/deutsch/ad-hoc-empfehlung-besondere-regeln-fuer-geimpfte.pdf). Konkret heißt es hierzu:

Aufgrund der Isolationsmaßnahmen sind die in Pflege‑, Senioren‑, Behinderten- und Hospizeinrichtungen Lebenden noch immer Belastungen ausgesetzt, die erheblich über das hinausgehen, was andere Bürgerinnen und Bürger erdulden müssen. Diese Sonderbelastung ist nur zu rechtfertigen, solange die in solchen Einrichtungen Lebenden noch nicht geimpft sind. Sie gehören unter anderem deshalb zur ersten Gruppe, die derzeit geimpft wird.

Bemerkenswerterweise blieben hier die spezifischen Gegebenheiten hinsichtlich der Bedürfnisse und Freiheitsrechte von im Maßregelvollzug untergebrachten Patienten – immerhin ein relevanter Teil aller in psychiatrischen Kliniken behandelter Patienten [11] – letztlich unberücksichtigt, obwohl sie ähnlichen Lebensbedingungen und Beschränkungen der Freiheits- und Besuchsrechte wie in Pflegeeinrichtungen untergebrachte Personen ausgesetzt waren und ebenfalls ein erhöhtes Sterberisiko aufweisen. Auch psychisch schwer erkrankte Patienten, die in allgemeinpsychiatrischen Versorgungsstrukturen behandelt werden, wurden im Rahmen der nationalen deutschen Impfstrategie zunächst nicht mit einer prioritären Impfempfehlung bedacht [4], obwohl sie einem nachweislich erhöhten Risiko ausgesetzt sind, infolge einer COVID-19-Erkrankung zu versterben [17]. Später wurde dann eine Priorisierung vergleichsweise schwerer erkrankter allgemeinpsychiatrischer Patienten empfohlen; schutzbedürftige Patienten im Maßregelvollzug erhielten jedoch bei anhaltender Knappheit der verfügbaren Impfstoffe weiterhin keine explizite Empfehlung zum bevorzugten Angebot einer COVID-19-Impfung. Die sich hieraus ergebende faktische Diskrepanz in der Behandlung psychisch erkrankter Menschen mutet angesichts der o. g. Befunde jedoch artifiziell an.

Im internationalen Vergleich ist erwähnenswert, dass Ende Dezember 2020 auch 47 % der Bundesstaaten der Vereinigten Staaten von Amerika keine spezifische Empfehlung dazu abgaben, wann unter Gefängnisbedingungen lebende Personen im Verlauf der Phasen der nationalen Impfkampagne ein Impfangebot erhalten sollten [22], obwohl deren erhöhtes Risiko infolge einer COVID-19-Erkrankung zu versterben bereits bekannt war [19].

Mit der Möglichkeit einer wirksamen Impfung gegen COVID-19 für im Maßregelvollzug untergebrachte Patienten verbindet sich aus der Sicht der Autoren nun einerseits die Hoffnung, dass Problemfelder, die einen direkten Bezug zur Pandemiesituation aufweisen, und eine Knappheit an Impfstoffen an Relevanz verlieren werden. Andererseits ergeben sich hierdurch möglicherweise auch neue Fragestellungen von praktischer Relevanz, die mittelfristig an Bedeutung gewinnen könnten.

## Einige relevante Problemfelder

### Einwilligungsfähigkeit

Während wohl kaum eine Debatte darüber entstehen dürfte, ob gemäß § 64 StGB untergebrachte Patienten mehrheitlich einwilligungsfähig hinsichtlich der Applikation einer Impfung gegen COVID-19 sein könnten, so stellt sich die Situation in der Maßregel gemäß § 63 StGB grundlegend anders dar, wo ein wesentlicher Teil der Untergebrachten an einer Schizophrenie erkrankt ist [10]. Insbesondere mit der Beurteilung der Einwilligungsfähigkeit in medizinische Maßnahmen sind jedoch hohe Anforderungen verbunden [5], denen Patienten mit chronischen, willensbestimmenden Krankheitssymptomen mitunter nicht gerecht werden. Doch weisen gerade an einer Schizophrenie erkrankte Patienten eine erhöhte COVID-19-Mortalität auf [13]. Gerade hier sind also eine engmaschige Zusammenarbeit mit gesetzlichen Betreuern und eine gründliche psychiatrische Befunderhebung im Hinblick auf die Einwilligungsfähigkeit der Patienten unbedingt geboten. Impfungen gegen den natürlichen Willen dieser überdurchschnittlich häufig Zwangsmaßnahmen unterworfenen Patientengruppe [18] erscheinen aus der Sicht der Autoren derzeit unter ethisch-moralischen Gesichtspunkten kaum begründbar. Andererseits kommt gerade Behandlern in der forensischen Psychiatrie eine wesentliche Rolle bei der Akzeptanz von Impfungen bei forensisch untergebrachten Patienten zu [1]. Dies setzt allerdings auch voraus, dass COVID-19-Impfungen unter Mitarbeitern der forensischen Psychiatrie eine hohe Akzeptanz aufweisen. Ferner wird die Frage zu beantworten sein, wie unter den Bedingungen des Maßregelvollzuges angesichts der bekannten Infektionsrisiken mit Patienten verfahren werden soll, die aufgrund einer freien Willensbildung eine COVID-19-Impfung ablehnen. Zu diesem Thema liegen mittlerweile erste Diskussionsbeiträge vor [12].

### Besondere Patientengruppen

Aktuelle Daten aus dem Vereinigten Königreich [9] weisen darauf hin, dass forensisch untergebrachte Patienten mit einem Migrationshintergrund, die auch in Deutschland im Vergleich zu ihrem Anteil an der Gesamtbevölkerung überdurchschnittlich häufig in forensisch-psychiatrischen Kliniken untergebracht sind [16], wahrscheinlich häufiger eine Impfung gegen COVID-19 ablehnen als andere Patienten. Hier wird in der Beratung und Aufklärung besonders auf kultursensible Aspekte der forensischen Therapie und etwaige Sprachbarrieren zu achten sein. Möglicherweise spielen auch Sozialisationsbedingungen in wirtschaftlich benachteiligten Regionen, Bürgerkriegsgebieten usw., wo Gesundheitsvorsorge und Impfungen gegebenenfalls eine geringere Bedeutung beigemessen wird, bei der Ablehnung einer COVID-19-Impfung eine Rolle. Es wird also darauf ankommen, eine im Vergleich schlechtere Beratung und Versorgung dieser Patientengruppe unbedingt zu vermeiden. In aktuellen Konsensuspapieren wird eine Verbesserung der Versorgung gerade dieser Patienten ausdrücklich angemahnt [14]. Möglicherweise könnte hier sprachunabhängiges Informationsmaterial dazu beitragen, Barrieren beim Zugang zu effektiven medizinischen Maßnahmen wie Impfungen zu reduzieren [6].

### Rehabilitationsperspektiven

Grundsätzlich erscheint auch die Frage berechtigt, inwiefern die Ablehnung einer COVID-19-Impfung oder eine diesbezüglich mangelnde Einwilligungsfähigkeit Auswirkungen auf die gesellschaftliche Rehabilitation außerhalb und die Behandlung dieser Patienten innerhalb forensischer Kliniken, beispielsweise in Bezug auf die Durchführung von Gruppentherapien, Lockerungen, Erprobungen und weitere vollzugsöffnende Maßnahmen, haben könnte. Derzeit werden für nicht gegen COVID-10 geimpfte Personen Beschränkungen des Zugangs zu bestimmten Aspekten des öffentlichen Lebens diskutiert und digitale Gesundheitsnachweise eingeführt. Hier wird einer möglichen Benachteiligung dieser Patienten oder einer Verzögerung des Behandlungsverlaufes im Zusammenhang mit Maßnahmen des Infektionsschutzes im Vergleich mit anderen Patienten, die eine Impfung erhalten haben, vorzubeugen sein. Darüber hinaus sind Auswirkungen auf die Möglichkeiten, Plätze in komplementären Wohneinrichtung mit dem Ziel der Durchführung einer Dauererprobung zu organisieren oder die Integration in einen Werkstattbetrieb zu ermöglichen, abzuwarten.

## Schlussbemerkung

Die oben aufgeführten Fragestellungen sind mitnichten als erschöpfend dargestellt zu betrachten, sondern sollen aus der Sicht der Autoren lediglich einen ersten Beitrag zur Diskussion spezifischer Fragestellungen im Hinblick auf COVID-19-Impfungen im Maßregelvollzug darstellen. Darüber hinaus sind viele weitere Fragen von direkter praktischer Relevanz denkbar: Wie sollte man beispielsweise damit umgehen, wenn Patienten bestimmte Impfstoffe ablehnen oder in einer spezifischen Klinik nur Impfstoffe zur Verfügung gestellt werden, die von der ständigen Impfkommission des Paul-Ehrlich-Instituts nicht für alle Altersgruppen empfohlen werden? Zusammenfassend zeigen sich hier also einmal mehr die großen Herausforderungen, denen Patienten und Behandler im Maßregelvollzug im Zusammenhang mit der COVID-19-Pandemie ausgesetzt sind.

Bisherige Erfahrungen an der Klinik für Forensische Psychiatrie des Pfalzklinikums weisen auf eine gute Annahme des Impfangebotes unter Patienten und Mitarbeitern hin (Stand Ende August 2021). Aufklärungs- und Beratungsgespräche mit an einer Schizophrenie erkrankten Patienten gestalteten sich erwartungsgemäß aufwendiger. Generelle, patientenunabhängige Schwierigkeiten hinsichtlich Beratung und Aufklärung ergaben sich daraus, dass noch Anfang Juni 2021 seitens der zuständigen Behörden keine Zusagen hinsichtlich des konkret zum Einsatz kommenden Impfstoffes erhältlich waren, was auch auf die eingangs erwähnte Knappheit der Impfstoffe und das Fehlen einer konkreten Priorisierungsempfehlung zurückzuführen gewesen sein mag.

Angesichts der eingangs skizzierten Befundlage wird im Zuge künftiger Pandemien also besonders auf die Bedürfnisse und Rechte marginalisierter Personengruppen, die unter den Bedingungen einer gerichtlichen Unterbringung leben, zu achten sein.

## Fazit für die Praxis

- Es liegen mittlerweile praktisch bewährte Empfehlungen zum Pandemiemanagement in der forensischen Psychiatrie vor.
- Bei der Beratung der Patienten sind deren Einwilligungsfähigkeit und Schutzbedürftigkeit unbedingt zu beachten.
- Behandler können die Entscheidung ihrer Patienten für eine Impfung dabei positiv beeinflussen.
- Patienten mit Migrationshintergrund sind im Hinblick auf den Zugang zu Impfungen möglicherweise benachteiligt.
- Rehabilitationsperspektiven von Patienten, die keine Impfung erhalten haben, müssen wo immer möglich gewahrt bleiben.
- Einer Benachteiligung forensischer Patienten muss im Zuge künftiger Pandemien bewusst entgegengewirkt werden.
